# The role of dung beetles in reducing greenhouse gas emissions from cattle farming

**DOI:** 10.1038/srep18140

**Published:** 2016-01-05

**Authors:** Eleanor M. Slade, Terhi Riutta, Tomas Roslin, Hanna L. Tuomisto

**Affiliations:** 1 Spatial Foodweb Ecology Group, Department of Agricultural Sciences, PO Box 27, Latokartanonkaari 5, FI-00014 University of Helsinki, Finland; 2 Department of Zoology, University of Oxford, South Parks Road, Oxford, OX1 3PS, United Kingdom; 3 Environmental Change Institute, School of Geography and the Environment, University of Oxford, South Parks Road, Oxford, OX1 3QY, UK; 4 Department of Ecology, Swedish University of Agricultural Sciences, Box 7044, 750 07 Uppsala, Sweden; 5 European Commission, Joint Research Centre (JRC), Institute for Environment and Sustainability, Via Enrico Fermi 2749, 21027 Ispra, Italy

## Abstract

Agriculture is one of the largest anthropogenic sources of greenhouse gases (GHGs), with dairy and beef production accounting for nearly two-thirds of emissions. Several recent papers suggest that dung beetles may affect fluxes of GHGs from cattle farming. Here, we put these previous findings into context. Using Finland as an example, we assessed GHG emissions at three scales: the dung pat, pasture ecosystem, and whole lifecycle of milk or beef production. At the first two levels, dung beetles reduced GHG emissions by up to 7% and 12% respectively, mainly through large reductions in methane (CH_4_) emissions. However, at the lifecycle level, dung beetles accounted for only a 0.05–0.13% reduction of overall GHG emissions. This mismatch derives from the fact that in intensive production systems, only a limited fraction of all cow pats end up on pastures, offering limited scope for dung beetle mitigation of GHG fluxes. In contrast, we suggest that the effects of dung beetles may be accentuated in tropical countries, where more manure is left on pastures, and dung beetles remove and aerate dung faster, and that this is thus a key area for future research. These considerations give a new perspective on previous results perspective, and suggest that studies of biotic effects on GHG emissions from dung pats on a global scale are a priority for current research.

Greenhouse gases are the biggest contributors to global warming and climate change^1^. GHG emissions from agriculture and associated land use change (LUC) were estimated to contribute 30% (8.0 Gt CO_2_e yr^-1^) of the global anthropogenic emissions in 2010^2^. Of GHG emissions from agriculture, livestock production accounts for around two-thirds (Fig. 1a), with direct emissions (4.6 Gt CO_2_e yr^−1^) emanating mainly from digestion by livestock^2^ (Fig. 1b). Dairy and beef production alone have been estimated to account for 60% of the total emissions of livestock production (Fig. 1c), with both enteric fermentation and fluxes from manure and its management being major contributors of GHGs^2^ (Fig. 1b).

Production of both meat and milk is projected to increase with a growing world population^3^. Grazing of beef and dairy livestock on grasslands, rather than intensive indoor production, can be used to mitigate livestock-related GHG emissions via the carbon sink of grass-fed production systems^4^. However, while grassland systems often act as sinks of CH_4_^5^,^6^, manure deposition may turn them from sinks to sources of GHGs^6^,^7^. Thus, the mitigation potential of manure management is an important issue for the livestock industry^3^,^8^.

Dung beetles (Scarabaeidae: Scarabaeinae, Aphodiinae, Geotrupidae) are some of the most important invertebrate contributors to dung decomposition in both temperate and tropical agricultural grasslands^9^,^10^,^11^,^12^,^13^. As such, they may help mitigate GHG emissions and aid carbon sequestration through removing dung deposited on the pastures, increasing grass growth and fertilization^14^. It has recently been shown that dung beetles have the potential to reduce GHG emissions from dung pats deposited on pastures^7^,^15^. Yet, how these effects compare to other GHG sources in livestock farming remains unknown, as the impact of dung beetles has not been included in more comprehensive lifecycle assessments of meat and dairy products.

In this study we use Finland as an example as this is one of the few regions of the world where all aspects of cattle farming and dung beetle impacts are well-documented. We adopt previously measured effects at the level of the dung pat to derive new estimates at the level the pasture ecosystem, and over the whole lifecycle of milk or beef production. By identifying the factors limiting the scope for dung beetle mitigation of GHG emissions in this highly-industrialized production system, we then point to other, still poorly-explored production systems in both temperate and tropical regions, where dung beetles are particularly likely to have an effect, and suggest that further research is urgently needed in these areas.

## Results

### Pat level emissions

**Figure 2:**
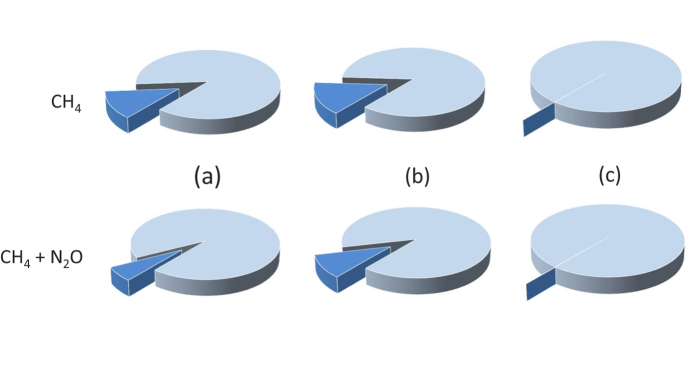
Reduction of GHG emissions by dung beetles at the level of (**a**) dung pats, (**b**) pastures and (**c**) the entire life cycle of beef and milk production. For (**a**), we show the reduction during the lifespan of a dung pat (59 days); for (**b**), we show mean daily flux from the pasture (including dung pats), and for (**c**), we refer to the entire life cycle of one kg of milk or one kg of meat. On the top row, we show effects on CH_4_ emissions, on the lower row we weight together the effects on CH_4_ and N_2_O fluxes as CO_2_ equivalents (see Methods for coefficients used).

At the level of the individual dung pat, dung beetles were found to reduce methane (CH_4_) emissions by 14.5% and nitrous oxide (N_2_O) emissions by 2.0%, resulting in an overall reduction of 7% in CO_2_ equivalents over the lifecycle of a pat (59 days) (Fig. 2a; Supplementary Material 2: Additional Results, Table S.1). This overall effect resulted from dung beetles significantly reducing CH_4_ fluxes from pats over the first 20 days of the pat lifetime, as compared with the fluxes from pats without dung beetles. After this period, the gas fluxes (both with and without beetles) stabilized to the same level as from the dung free pasture (treatment x day, F_7,217_ = 15.87, P < 0.001). Dung beetles had no significant effect on the N_2_O fluxes (treatment x day, F_7,217_ = 0.65, P = 0.71) (Supplementary Material 2: Additional Results, Fig. S.1).

### Pasture level emissions

**Figure 3:**
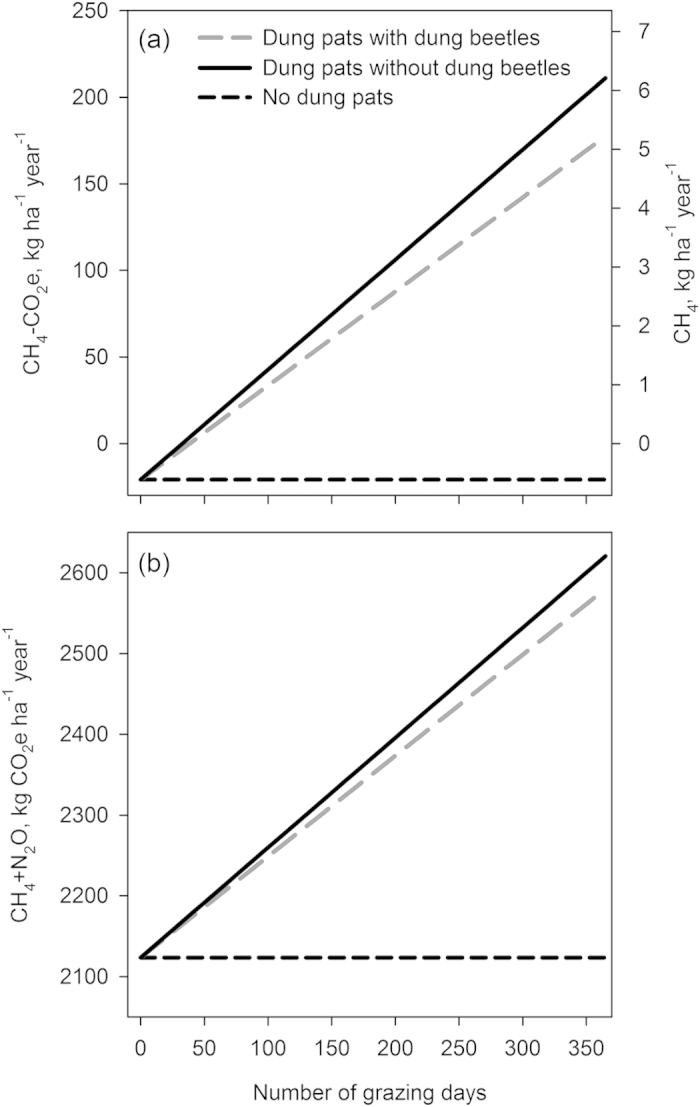
The effect of the grazing season length on the annual fluxes of (**a**) CH_4_ and (**b**) CH_4_ and N_2_O combined (as carbon dioxide equivalents, 100 year time horizon) from a pasture with dung pats and dung beetles, with dung pats but no dung beetles, and no dung pats. Negative values indicate an ecosystem sink and positive values indicate a source to the atmosphere.

At the level of the pasture, dung beetles also had a substantial effect on total GHG fluxes. Overall, emissions from dung offset the CH_4_ sink of the pasture soil, turning the pasture from a modest CH_4_ sink into a source. However, the presence of dung beetles reduced the mean daily CH_4_ emissions during the grazing season by 17% as compared with the situation where no dung beetles were present. On an annual scale, taking into account the non-grazing period, the dung beetle effect on the pasture CH_4_ fluxes was 21% (Fig. 2b). Dung beetles slightly reduced the N_2_O fluxes from the pasture-by 5% during the grazing season and by 0.1% over the course of the year (Supplementary Material 2: Additional Results, Table S.2). In terms of the two gases combined into CO_2_ equivalents (see Methods for details), dung beetles reduced the emissions by 12% during the grazing season and by 0.5% annually (Supplementary Material 2: Additional Results, Table S.2). Clearly, as fluxes from dung pats are a major component of the total fluxes from a pasture, the pasture-level GHG balance is strongly dependent on the number of grazing days (Fig. 3). With a longer grazing period, overall CH_4_ and N_2_O emissions increase, and so does the relative mitigating effect of the dung beetles (Fig. 3).

### Lifecycle of meat and milk production emissions

At the level of the full life cycle of meat and dairy products, the reduction of GHG emissions by dung beetles accounts for only 0.08% of the total life cycle assessment based GHG emissions of milk when Land Use and Land Use Change (LULUC) related emissions are not included, and for 0.05% when LULUC emissions are included. For beef, the reductions are 0.13% when LULUC emissions are not included and 0.07% when LULUC emissions are included (Table 1, Fig. 2c). This is due to the fact that total N_2_O and CH_4_ emissions from dung pats deposited on grazing land account for only 1–4% and 0.1–0.4% respectively, of the total GHG emissions of milk and beef production (see Table 1).

## Discussion

If mitigation techniques are not implemented then greenhouse gas emissions from agriculture are projected to rise to 8.2 billion tonnes of CO_2_ equivalents by 2030^3^. Manure-related sources are also projected to increase during this time period – N_2_O by 29% and CH_4_ by 20%^3^. The potential for mitigation of these emissions is huge, but at present few techniques are cost effective^3^,^4^. One way of reducing GHG emissions from cattle farming for milk and beef is to graze the livestock on open grassland, rather than using intensive grain fed systems^4^. However, this study and others^6^,^7^, show that dung additions to a pasture can turn it from a sink to a source of CH_4_. Dung beetles may help mitigate these effects through the aeration and burial of dung pats^7^. In this paper, we assessed the contribution of dung beetles to reduction of GHG emissions in Finland at three nested scales – the dung pat, the pasture ecosystem and the whole lifecycle of milk or beef production. At the lower two levels, dung beetles were found to play an important role in reducing GHG emissions: during the grazing season, beetles reduced GHG emissions from pats and pastures by up to 7% and 12%, respectively, mainly through large reductions in CH_4_ emissions. Yet, over the full lifecycle of beef and milk production, the impact of dung beetle mediated effects was dwarfed by other impacts, suggesting a limited net impact in the context of highly-intensive production systems such as that of Finland. Below, we address each finding in turn.

At the pat level, dung beetles significantly reduced fluxes of CH_4_, with less-consistent effects on N_2_O. As proposed by Penttilä *et al.*^7^, the effect on CH_4_ fluxes can likely be traced to an oxygenating effect on the dung pat interior: As CH_4_ is formed under anaerobic conditions, holes dug by the beetles may enhance the drying of dung pats and increase the availability of oxygen in the deeper parts of the pats, thus increasing aerobic decomposition, decreasing anaerobic decomposition and reducing methanogenesis^16^.

At the pasture level, CH_4_ production in dung pats makes them ‘hotspots’ for GHG fluxes as compared to pasture without dung. Thus, any changes in the fluxes from these pats may result in large relative changes for the whole pasture – although the absolute changes may be fairly small. Taking into account the non-grazing season (approximately 250 days in Finland), during which no new dung pats are added and emissions from previous dung pats approach zero, dung beetles contribute an annual reduction of 21% in CH_4_ emissions and 0.4% in N_2_O and CH_4_ emissions combined, per one hectare of pasture. The amount of actively grazed pasture needed to sustain the current Finnish cattle stock of 1 million heads (assuming that around 80% of them graze outside^17^), is approximately 960 km^2^ (pasture land calculator, which incorporates default local conditions, https://portal.mtt.fi/portal/page/portal/Artturi/Artturikirjasto/Laskurit/Laidunalan_hallinta). Over such an area, dung beetles can reduce the annual country-scale emissions of CH_4_ and N_2_O by 900 t of CO_2_ equivalents, compared to a situation where no beetles were present. This effect size equals 0.12% of total emissions from manure management systems (0.72 Mt CO2e^18^). However, the amount of dung deposited on pastures, and therefore the fraction of manure on which dung beetles may act, is naturally proportional to the length of the grazing season (see Fig. 3). This period is short in Finland, but much longer in many countries in Central and Southern Europe^19^ (in southern Italy, cattle may be kept outdoors the whole year round). Thus, mitigation of GHGs using dung beetles becomes a more efficient strategy the longer cows are grazed on pastures. Longer grazing seasons may also benefit dung beetle populations^20^, increasing the abundance and diversity of dung beetles in the pasture, and hence increasing dung removal over the long term. It is also important to note that the relative dung beetle effect on the total pasture flux is dependent on the GHG fluxes and the CH_4_ sink strength from the dung-free parts of the pastures, and may vary with several biotic and abiotic factors^6^ (see Supplementary Material 3: Sensitivity Analysis). However, although potential changes in fluxes, due for example to changes in abiotic conditions, such as rainfall and temperature, will change the absolute flux estimates for the total pasture (dung pats and dung free areas), we expect the dung beetle effect to remain relatively stable. Our sensitivity analysis shows that this is the case and that particularly at the LCA level the total dung beetle effect changes little with simulated changes to fluxes (see Supplementary Material 3: Sensitivity Analysis).

At the level of the entire life cycle of milk and beef production, dung beetles in Finland reduced GHG emissions by 0.05–0.13% depending on whether LULUC emissions are included or not. Thus, the contribution of the emissions from dung pats deposited on pasture land is dwarfed in comparison to other emissions of milk and meat production, such as methane emissions from enteric fermentation, nitrous oxide emissions from soils, and carbon dioxide emissions from energy use^2^. As a consequence of limited outdoor livestock grazing, and intensive indoor production of beef and milk, European emissions from manure left on pasture account for only 6% of the direct agricultural emissions^2^. The key factors constraining dung beetle mediated effects in the intensive farming systems of temperate Europe are thus the length of the grazing period, the fraction of cattle grazing under outdoor conditions – and naturally, the functional efficiency and seasonality of the dung beetles. Thus, the contribution of different emission sources to the total GHG emissions of dairy farming may differ among different countries in Europe^21^,^22^, and also between different dairy farming management practices^23^. While these factors may lead to changes in fluxes, we expect the total effect of dung beetles to remain relatively stable and minor, compared with the other emissions from the production of milk and beef. Thus, the qualitative effects of dung beetles on GHG emissions are comparatively large at the pasture level, but markedly small when assessed over the entire lifecycle of beef or milk production in Finland (see Supplementary Material 3: Sensitivity Analysis).

The relative contribution of dung beetles to mitigating GHG emissions will likely differ between regions. Given substantial differences in both cattle management and dung beetle ecology between different parts of the world^23^,^24^, the effects of dung beetles on GHG emissions are likely to vary both within and between latitudes. However, some clear-cut contrasts between regions can be identified: In regions where outdoor livestock grazing is more commonly used, the emissions from manure left on pasture will have a larger contribution to total agricultural emissions, with estimated fractions ranging from 11% in Asia up to 35% in Africa^2^,^25^,^26^. Such patterns are combined with likely differences in dung beetle efficiency: In tropical regions, dung beetles can remove the majority of a fresh dung pat within the first few days after deposition^12^,^13^,^27^ – whereas in temperate conditions, a substantial fraction will remain throughout the grazing season^11^. The high CH_4_ fluxes from fresh pats, which under temperate conditions occur within the first 10–20 days of the pat being deposited^7^ (Supplementary Material 2: Additional Results, Fig. S.1), may thus be effectively avoided in the presence of tropical dung beetles. As a key avenue for future research, we thus suggest that contributions of dung beetles to reductions in GHG emissions should be partitioned under tropical conditions. While latitudinal patterns in average conditions may be compounded by finer-scale variation in microclimate^28^, we make a clear-cut and testable prediction: that effects at all levels from dung pats through pastures to the whole lifecycle of milk or beef production may be strongly accentuated at low latitudes.

Worryingly, dung beetles have been declining rapidly throughout temperate and tropical ecosystems due to changes in agricultural practices, including intensification and reduced pasture grazing^29^,^30^,^31^, habitat loss^32^,^33^, and overuse of anthelmintics (dewormers), such as ivermectins^34^,^35^. In Finland, over half the dung beetle fauna is now considered extinct, endangered or near threatened^20^,^36^. The knock-on effects of these declines for ecosystem functioning and the many services that dung beetles provide has been highlighted by recent papers, showing that dung removal services may be reduced when dung beetle communities change under fragmented environments or through ivermectin use^34^,^37^. Thus, the mitigation of GHG fluxes at the pat and pasture level is a further service provided by dung beetles, and is now potentially threatened by continuing biodiversity loss.

## Methods

In Finland, emissions from the agricultural sector, agricultural soils, and agricultural energy use comprise 21% of total GHG emissions^18^. The country has a large milk and beef industry, with almost 1 million heads of cattle, producing an annual total of 4 billion kg of dung^38^. We use Finland as an example, as this is one of the few regions of the world where local and/or regional estimates of GHG emissions^18^, cattle farming^2^,^39^, and dung beetle-mediated effects^7^ (and current study) are readily available. Thus this offers a unique opportunity to derive estimates of dung beetle contributions at levels from dung pats to beef and milk production.

### Dung beetle data

To quantify the contribution of dung beetles to gas fluxes from individual dung pats, we used a large-scale mesocosm experiment. As the number of species encountered per natural dung pat in boreal and temperate regions is typically low (median 2 species per pat, range 1–8 in a sample of 797 dung pats from across Finland (taken from Roslin)^40^), we constrained our experiments to relatively small species pools. Hence, communities of varying richness and relative abundance were constructed using four common early summer north temperate dung beetle species (*Geotrupes stercorarius* (Linnaeus, 1758), *Aphodius erraticus* (Linnaeus, 1758), *Aphodius pedellus* (DeGeer, 1774), and *Aphodius fossor* (Linnaeus, 1758)), with the abundances of each species representing realistic abundances observed in the field^32^,^37^. Dung beetle abundances are highest in the early summer^20^. Hence, any functional effects of the beetles are expected to be smaller for the pats deposited later during the grazing season, making the current estimates of dung beetle effects on the high side.

Dung beetles were collected from the pastures of the Koskis Manor in Salo, Southwestern Finland (60°22′49″N 23°17′39″E) and Karjalohja (60°11′28″N 23°40′19″E) between 5 and 7^th^ June 2012. Beetles were stored in mixed sex groups in moist paper at +5 °C, until being assigned randomly to treatments. All dung was manually homogenized before partitioning into experimental pats of 1.2l within 5 hours of collection. For details on the dung used (see Supplementary Material 1: Additional Methods).

The mesocosms (n = 36) used were constructed from plastic buckets with their bottoms sawn off (cylinder 58 cm in diameter at ground level, height 32 cm, and inserted 20 cm into the ground). Fine environmental mesh (1 mm) covered the tops of the mesocosms, to prevent the beetles escaping. The mesocosms were opened after 20 days to allow the beetles to emigrate rather than force them to artificially inhabit the pat.

The experiment was carried out on a grass sward reflecting a multiannual Finnish pasture, located in Viikki, Helsinki, Southern Finland (60° 13′ 31″ N 25° 1′ 0″ E). On the 8th June 2012 dung and beetles were added to 30 of the mesocosms, three of the mesocosms received only dung and a further three were grass only controls and received neither dung nor beetles. The experiment was run for 60 days as this corresponds to the adult and larval lifecycle of the beetles and the lifecycle of the dung pat from fresh to dry and decayed^11^. Vegetation inside was kept low by manual trimming.

To evaluate gas fluxes from the dung pats, we used a static closed chamber method^41^. The procedures used followed those described in Penttilä *et al.*^7^ and are briefly summarised in Supplementary Material 1: Additional Methods. Measurements of N_2_O and CH_4_ were conducted on days 1, 3, 6, 10, 14, 20, 30, 40, 59 and cumulative fluxes calculated separately for each chamber as areas under the temporal gas flux curve^7^. After 59 days, the emissions from the pats had subsided and the pat was decomposed. Therefore, when calculating fluxes, we consider 59 days as the lifetime of a pat.

To evaluate the overall warming effect of GHG emissions from dung pats, compound-specific emissions should be gauged against each other. GHG emissions from dung pats are generally dominated by CO_2_^7^. However, previous work suggests the main mitigation potential of dung beetles to reside in effects on N_2_O and CH_4_^7^, so we chose to focus on these specific compounds. To weigh their fluxes together in a joint currency, we converted them to CO_2_-equivalents by using the IPCC 2013 global warming potential (GWP) impact factors for 100 years’ time period, 298 for N_2_O and 34 for CH_4_^42^.

The pat-scale contribution of dung beetles to the fluxes was assessed by comparing the fluxes from dung pats with and without dung beetles. A generalized linear mixed-effects model was fitted to daily flux values, with treatment (dung beetles present or absent) and measurement day as fixed effects, and mesocosm as a random effect. To account for the temporal dependence of the consecutive measurements, we applied a first order autoregressive correlation structure to each mesocosm. To account for the heterogeneity of variance between treatments, we applied a separate variance structure for each treatment where necessary. Separate models were fitted to data on CH_4_ and N_2_O fluxes. The analyses were carried out using the nlme package^43^ of R^44^.

### GHG flux estimates for the pasture ecosystem

The effect of dung beetles on GHG fluxes at the pasture level was quantified using the data described above on fluxes from dung pats with and without dung beetles. Following Saarijärvi *et al.*^45^, we assumed that 4% of the active pasture area was covered by dung pats at any given time during the grazing season, and that the grazing season lasted 110 days. Since the dung pats in real pastures will be of different ages, and in different stages of decomposition, we used the average flux measured over the 59 days in our calculations. The total grazing season pasture flux was then calculated as a sum of the flux from dung pats (with or without dung beetles) and the flux from pasture without pats, multiplied by their respective area proportions.


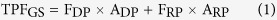


where TPF_GS_ is the total pasture flux during the growing season, F_DP_ is the flux from dung pats (calculated with and without dung beetles), A_DP_ is the proportion of area covered by dung pats, F_RP_ is the flux from the residual pasture (no dung pats), and A_RP_ is the proportion of the area of the residual pasture. A detailed breakdown of these calculations, including formulas used, are presented in Supplementary Material 4: Flux Calculations.

For pasture-level fluxes outside the grazing season, we used values for the residual pasture (dung-free areas) from the literature with year-round, multi-year data^6^,^46^. Outside the grazing season, we assumed that no flux was coming from dung pats, old pats having been decayed and no new pats deposited. Annual flux was calculated as the product of the daily mean flux during the grazing season (derived from Eq.1) and the number of grazing days, summed with the product of the daily mean flux during the non-grazing season and the number of non-grazing days.


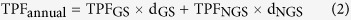


where TPF_annual_, TPF_GS_ and TPF_NGS_ denotes the total annual, grazing season and non-grazing season pasture flux, respectively, and d_GS_ and d_NGS_ denote the length of grazing and non-grazing seasons (number of days).

As the annual estimates are sensitive to the grazing season length, which is likely to vary geographically, between years and depending on management practices, we also carried out a sensitivity analysis, varying the grazing season length from 0 to 365 days.

### Life cycle assessment data

Life cycle assessment (LCA) is a method used for assessing the environmental impacts of a product or service during the whole production chain from extraction of raw material up to waste management^47^. So far, the contribution of dung beetles in reducing GHG emissions from milk and beef production has not been taken into account in milk and beef LCAs. We used the results from the field experiments described in the section ‘Dung beetle data’ above to incorporate the dung beetle effect in to the LCAs; namely, the beetle-mediated reduction of CH_4_ and N_2_O emissions from dung pats deposited on pasture land. The dung beetle effect was calculated by multiplying the emissions from grazing animals in Table 1 by the proportional emission reductions by dung beetles in Supplementary Material 2: Additional Results, Table S.2.

The general LCA data for milk and beef produced in Finland was based on data from Leip *et al.*^39^. Their study considered (i) on-farm livestock rearing including enteric fermentation, manure deposition by grazing animals, manure management and application of manure to agricultural land; (ii) fodder and feed production including application of mineral fertiliser, the cultivation of organic soils, crop residues and related upstream industrial processes (fertilizer production); (iii) on-farm energy consumption related to livestock and feed production and energy consumption for the transport and processing of feed; (iv) land use changes induced by the production of feed (excluding grassland and grazing); and (v) emissions (or removals) from land use through changes in carbon sequestration rates related to feed production (including grassland and grazing). Leip *et al.*^39^ also included a scenario without considering the Land Use and Land Use Change (LULUC) related emissions and three scenarios including the LULUC emissions. Those three scenarios varied with respect to what type of land was converted to agricultural land. Scenario I assumed that all additional cropland was converted from grassland and savannas. Scenario II assumed more likely mix of land types converted to agricultural land, whereas scenario III includes a high share of converted forests.

Based on the data from Leip *et al.*^39^ and with adjustment to the IPCC 2013 emissions factors^48^, the average GHG emissions of milk and beef meat production in Finland are 1.30 kg CO_2_e/kg milk and 21.76 kg CO_2_e/kg beef meat when the LULUC related emissions were not taken into account. When the LULUC emissions were taken into account, the emissions varied between 2.08–2.17 kg CO_2_e/kg milk and 39.98–41.33 kg CO_2_e/kg beef meat depending on the scenario. In this study we used the scenario without LULUC emissions and the Scenario II with LULUC emissions. The breakdown of the emissions relevant for this study is presented in Table 1 and a more detailed breakdown of the emission calculations, with formulas, is presented in Supplementary Material 1: Additional Methods and Flux Calculations. As above, the N_2_O and CH_4_ emissions were converted to CO_2_-equivalents by using the IPCC 2013 global warming potential (GWP) impact factors for 100 years’ time period, 298 for N_2_O and 34 for CH_4_^42^. Leip *et al.*^39^ report the N_2_O emissions from dung pats deposited on pasture land as a category separate from other manure management, calling it ‘grazing animals’ (Table 1). However, the CH_4_ emissions from dung pats are included in the general category ‘manure management’, which includes other manure management methods. Therefore, further calculations were needed to estimate the CH_4_ emissions from dung pats (See Supplementary Material 1: Additional Methods). Based on these calculations the total CH_4_ emissions from dung pats deposited on grazing land were 0.00348 kg CO_2_e/kg milk and 0.0853 kg CO_2_e/kg meat (Table 1).

## Additional Information

**How to cite this article**: Slade, E. M. *et al.* The role of dung beetles in reducing greenhouse gas emissions from cattle farming. *Sci. Rep.* **6**, 18140; doi: 10.1038/srep18140 (2016).

## Supplementary Material

Supplementary Information

Supplementary Material

## Figures and Tables

**Figure 1:**
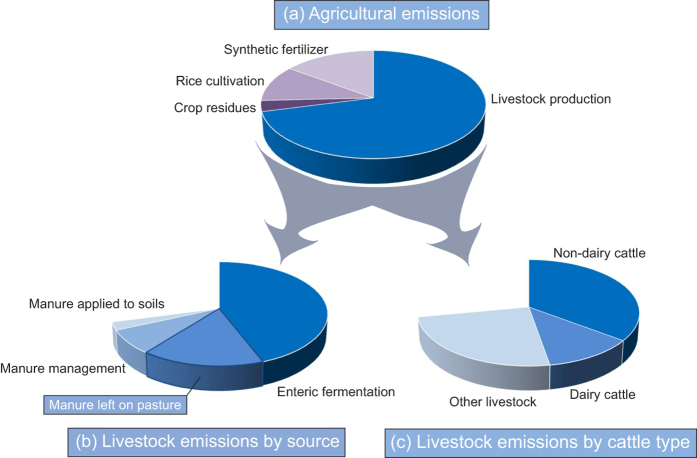
Global contributions to direct greenhouse gas emissions from agriculture. Shown are (**a**) contributions to all direct agricultural emissions (grand total 4.6 Gt CO_2_e yr^−1^), with emissions from livestock production split further in two alternative ways – according to (**b**) different types of emissions, and (**c**) different types of livestock. Sources for (**a**,**b**): Tubiello *et al.*^2^; for c: http://faostat3.fao.org/browse/G1/*/E.

**Table 1:** Breakdown of the life cycle assessment based greenhouse gas emissions of milk and beef produced in Finland.

	Milk	Beef
**Total GHG emissions**		
Without LULUC	1.30	21.76
With LULUC	2.10	40.20
**N**_**2**_**O emissions**		
Total N_2_O	0.454 (35, 22)	7.30 (36, 19)
Manure management	0.029 (2, 1)	0.494 (2, 1)
Grazing animals	0.026 (2, 1)	0.763 (4, 2)
Change by dung beetles^a^	−0.0005 (0.04, 0.02)	−0.0153 (0.07, 0.04)
**CH**_**4**_ **emissions**		
Total CH_4_ emissions	0.597 (46, 28)	9.982 (46, 25)
Manure management^b^	0.065 (5, 3)	0.464 (3, 1)
Grazing animals^c^	0.003 (0.2, 0.1)	0.085 (0.4, 0.2)
Change by dung beetles^a^	−0.0005 (0.04, 0.02)	−0.0123 (0.06, 0.03)
**Total N2O + CH4 emissions**	0.0010 (0.08, 0.05)	0.0278 (0.13, 0.07)

N_2_O values were taken from Leip *et al.*^39^ and CH_4_ figures were calculated in Supplementary Material 1: Additional Methods. Values are in kg CO_2_e/kg_milk/meat_. The proportional contribution (%) of the different emissions sources to the total emissions of milk and beef production is shown in brackets, excluding and including LULUC emissions, respectively. The CH_4_ emissions are modified with the new IPCC 2013 global warming potential emission factor 34^42^. The ‘Change by dung beetles’ values show the reduction in the emissions from grazing animals when the dung beetle effect is taken into account.

^a^Calculated by multiplying the emission from grazing animas by the dung beetle effect (see Supplementary Material 4: Flux Calculations).

^b,c^Leip *et al.*^39^ reported total manure management (including grazing animals) emissions for CH_4_ (0.068 and 0.549 kg CO_2_e for kg of milk and beef, respectively), which we partitioned into emissions from manure management excluding grazing animals and emissions from grazing animals, as described in Supplementary Material 1: Additional Methods.
