# Erratum: The role of dung beetles in reducing greenhouse gas emissions from cattle farming

**DOI:** 10.1038/srep22683

**Published:** 2016-03-08

**Authors:** Eleanor M. Slade, Terhi Riutta, Tomas Roslin, Hanna L. Tuomisto

Scientific Reports 6: Article number: 1814010.1038/srep18140; published online: 01052016; updated: 03082016

The original version of this Article contained an error in the Abstract.

“These considerations give a new perspective on previous results perspective, and suggest that studies of biotic effects on GHG emissions from dung pats on a global scale are a priority for current research.”

now reads:

“These considerations give a new perspective on previous results, and suggest that studies of biotic effects on GHG emissions from dung pats on a global scale are a priority for current research.”

This has now been corrected in the PDF and HTML versions of the Article.

