# Supplementary material for: The role of dung beetles in reducing greenhouse gas emissions from cattle farming
Source: Sci Rep. 2016 Jan 5;6:18140. doi: 10.1038/srep18140 (PMC4700445; doi:10.1038/srep18140)
Supplement: Supplementary Information [file srep18140-s1.pdf]

Supplementary Material

The role of dung beetles in reducing greenhouse gas emissions from cattle farming

Eleanor M. Slade[a,b]*, Terhi Riutta[c], Tomas Roslin[a,d], Hanna L. Tuomisto[e]

[a] Spatial Foodweb Ecology Group, Department of Applied Biology, PO Box 27, Latokartanonkaari 5, FI-00014 University of Helsinki, Finland. eleanor.slade@zoo.ox.ac.uk

[b]Department of Zoology, University of Oxford, South Parks Road, Oxford, OX1 3PS, United Kingdom

[c]Environmental Change Institute, , School of Geography and the Environment, University of Oxford, South Parks Road, Oxford, OX1 3QY, UK. terhi.riutta@ouce.ox.ac.uk

[d]Department of Ecology, Swedish University of Agricultural Sciences, Box 7044, 750 07 Uppsala, Sweden

[e]European Commission, Joint Research Centre (JRC), Institute for Environment and Sustainability, Via Enrico Fermi 2749, 21027 Ispra, Italy. hanna.tuomisto@jrc.ec.europa.eu

*corresponding author: Department of Zoology, University of Oxford, South Parks Road, Oxford, OX1 3PS, United Kingdom. eleanor.slade@zoo.ox.ac.uk. Tel: +44 (0)1865 271163

Supplementary Material 1: Additional Methods

Details on the dung used in the experiments

Fresh, unmedicated cattle dung was obtained from the Viikki Study and Research Farm, owned by the University of Helsinki. The dung was collected from a herd of 20 adult dairy Ayrshire cattle. The cattle had been grazing daily for approximately a month on improved pastures sown with a mix of timothy (*Phleum pratense*) and meadow fescue (*Festuca pratensis*) with a smaller component of red clover (*Trifolium pratense*). Outdoor grazing time ranged from 4 to 5 hours per day between 8 AM and 2 PM, with the dung collected as the cattle entered the barn for within-stall milking. When indoors, the cattle was fed additional silage, a standard concentrate (Maituri 20 and Aminomaituri 30, Raisio Oyj, Raisio, Finland), and magnesium-selenium minerals (Pihatto-Melli; Raisio Oyj, Raisio, Finland). No animal in the herd had been given antibiotics or antiparasitic treatments.

Methods used in flux measurements

Gas samples were taken with a syringe after 5, 10, 20, and 30 minutes of the chamber being sealed, and injected into glass vials (3-ml Labco Exetainers® with double septa, Labco Ltd., Buckinghamshire, UK). CH_4 and N_2O were then quantified in parts per million (ppm by volume) by gas chromatographs (HP 5890 Series II, Hewlett Packard, Palo Alto, CA, U.S.A.) equipped with thermal conductivity, flame ionization and electron capture detectors. Ambient air temperature in the shade, next to each chamber at 20 cm height, was recorded during the sampling of all gases, for later scaling of gas fluxes to temperatures. For further details, see Penttilä et al.[1].

Calculation of CH_4 emissions from grazing animals

We used data about the proportion of each manure management system used in Finland and the methane producing capacity of the manure management systems (Eq. 1).

$$ME_g = MPF_g \times P_g \times \frac{ME_m}{\sum(MPF_i \times P_i)} \qquad (1)$$

where

ME_g = *methane emissions from manure deposited on grazing land*

ME_m = *methane emissions from manure management in total*

MPF_i = *Methane production fraction from manure management system i*

P_i = *Proportion of manure management system i*

i = Manure management system (g, l, s)

g = grazing system

l = liquid manure system

s = solid manure system

Based on data from Leip et al.[2], 20% of the manure from dairy cows and 35% from beef cattle is deposited on pasture land in Finland, whereas the proportions of manure managed in liquid and solid systems are 36% and 44% for dairy cows and 16% and 49% for beef cattle, respectively. Based on Leip et al.[2], and with adjustment to the new CH4 emission factor[3], the following methane production fractions were used for the respective manure management systems: 0.177 for liquid manure, 0.027 for solid storage and 0.020 for manure deposited on grazing land.

References

1 Penttilä, A. *et al.* Quantifying Beetle-Mediated Effects on Gas Fluxes from Dung Pats. *PLoS ONE* **8**, e71454, doi:10.1371/journal.pone.0071454 (2013).

2 Leip, A. *et al. Evaluation of the livestock sector's contribution to the EU greenhouse gas emissions (GGELS) - final report*. (European Commission, Joint Research Centre, 2010).

3 Myhre, G. *et al.* Anthropogenic and natural radiative forcing, Chapter 8 in Climate Change 2013: The Physical Science Basis. Contribution of Working Group I to the Fifth Assessment Report of the Intergovernmental Panel on Climate Change, edited by Stocker, TF, D. Qin, G. *K. Plattner, M. Tignor, SK Allen, J. Boschung, A. Nauels, Y. Xia, V. Bex and PM Midgley, Cambridge University Press, Cambridge, UK and New York, NY* (2013).

Supplementary Material 2: Additional results.

As our paper focuses on estimating the relative contribution of dung beetle mediated effects on GHG emissions at the level of dung pats, pastures and the life cycle of beef and milk production, we focus the presentation in the main paper on these proportions. Here, we give the detailed analyses and specific numbers underlying these proportions. The step-by-step calculations for these estimates are provided in an Excel spreadsheet, Supplementary Material 4: Flux Calculations.

Table S.1. Mean daily fluxes (mg of CH_4, N_2O or CO_2e m^{-2} day^{-1}) ± SE of CH_4 and N_2O from dung pats over the 59 day experimental period (with day 0 equaling the fresh pat). Negative values indicate an ecosystem sink and positive values indicate a source to the atmosphere.

	CH_4	CH_4-CO_2e	N_2O	N_2O-CO_2e	CH_4+N_2O, CO_2e
Pat with dung beetles (DB)	39.8 (±1.22)	1353 (±30.6)	7.91 (0.641)	2356 (±191)	3709 (±210)
Pat without dung beetles (C)	46.5 (±3.73)	1581 (±93.4)	8.07 (±1.66)	2404 (±496)	3986 (±581)
Dung-free pasture	-0.310 (±0.194)	-10.5 (±4.85)	-0.197 (±0.188)	-58.6 (±55.9)	-69.1 (±57.9)
Dung beetle effect, %[a]	-14.5	-14.5	-1.98	-1.98	-6.95

[a] Calculated as ((DB - C)/C)*100

Table S.2. CH_4 and N_2O fluxes from pasture land, with and without dung beetles, during the grazing season (GS) and annually. The calculations are based on the assumption that the grazing season lasts 110 days during which time dung pats cover 4% of the pasture area. The fluxes reported are weighed by the proportion of their source area: dung pats (with or without dung beetles) 4%, the pasture not covered by pats 96% (100% outside grazing season), and the pats and pasture combined (total pasture) 100%. The units are kg of CH_4, N_2O or CO_2e ha^{-1} $year^{-1}$. Negative values indicate an

ecosystem sink and positive values indicate a source to the atmosphere. Note that for the dung pats (with and without dung beetles), the values given for the grazing season also represent annual values, because it is assumed that there are not dung pats present outside the grazing season.

Flux source area	CH_4	CH_4-CO_2e	N_2O	N_2O-CO_2e	CH_4+N_2O, CO_2e
Dung pats, with dung beetles, GS	1.75	59.5	0.35	104	163
Dung pats, no dung beetles, GS	2.05	69.6	0.35	106	175
Dung-free areas of pasture, GS	-0.327	-11.1	-0.208	-61.9	-73.0
Dung-free areas of pasture, annual	-0.605[a]	-20.6	7.11[b]	2118	2098
Total pasture, GS, with dung beetles (DB_{GS})	1.42	48.1	0.140	41.8	90.2
Total pasture, GS, no dung beetles (C_{GS})	1.72	58.5	0.147	43.9	102
Total pasture, annual, with dung beetles (DB_{annual})	1.15	39.0	7.46	2222	2261
Total pasture, annual, no dung beetles (C_{annual})	1.44	49.1	7.46	2224	2273
Dung beetle effect, GS (%)[c]	-17.3	-17.3	-4.78	-4.78	-11.9
Dung beetle effect, annual (%)[d]	-20.6	-20.6	-0.094	-0.094	-0.537

[a] From the multi-year study by Maljanen et al.[1], with year-round measurements
[b] From the multi-year study by Virkajarvi et al.[2], with year-round measurements
[c] Calculated as $((DB_{GS} - C_{GS})/C_{GS})*100$
[d] Calculated as $((DB_{annual} - C_{annual})/C_{annual})*100$

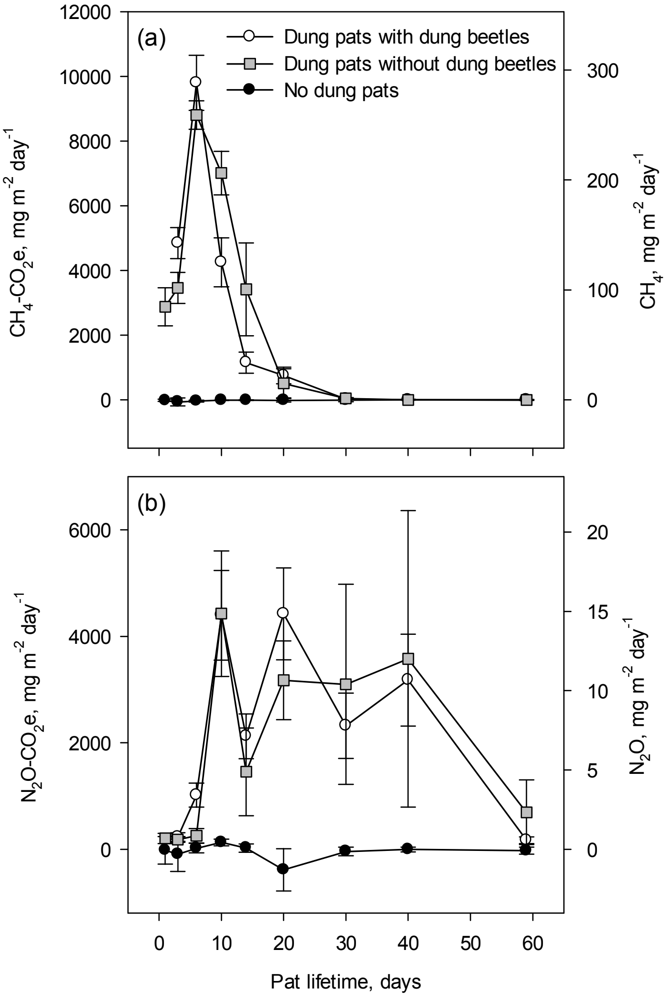

Figure S.1. Measured fluxes of a) methane (CH_4) and b) nitrous oxide (N_2O) from mesocosms containing dung pats with dung beetles, dung pats without dung beetles, and no dung. The pats were fresh at day 0. Error bars are 95% confidence intervals.

References

1 Maljanen, M. E., Virkajärvi, P. & Martikainen, P. J. Dairy cow excreta patches change the boreal grass swards from sink to source of methane. *Agriculture and Food Science* **21**, 91-99 (2012).

2 Virkajärvi, P., Maljanen, M., Saarijärvi, K., Haapala, J. & Martikainen, P. J. N2O emissions from boreal grass and grass - clover pasture soils. *Agriculture, Ecosystems & Environment* **137**, 59-67, doi:http://dx.doi.org/10.1016/j.agee.2009.12.015 (2010).

Supplementary Material 3: Sensitivity analysis

To examine which pasture flux components (dung pats, contribution of the beetles, dung-free areas of the pasture) had the largest influence in the results, we carried out a sensitivity analysis, where we varied the length of the grazing season, the flux from the dung-free areas of the pasture, the flux from dung (dung flux rate varied, but beetle effect kept constant), and the contribution of the dung beetles. Such variation may be due to various abiotic (e.g. temperature, moisture, climate, soil type) and biotic (e.g. dung beetle abundance and community composition, vegetation type) factors. As response variables, we used the total annual flux of the pasture (which could have dung pats with or without dung beetles in it, or be devoid of dung) and the emissions over the life cycle of milk and beef production. For the LCA sensitivity analysis, we chose the scenario without LULUCF emissions (see Table 1 and Leip et al (2010)[1]).

For all but grazing season length, the range of values used for the explanatory variables were derived using the value observed / calculated in this study as a starting point, ± three times the value as the upper and lower ranges. The length of the grazing season was allowed to vary from 0 to 365 days per year.

The annual CH_4 flux at the pasture scale was most sensitive to the variation in grazing season length and flux rate from dung pats, while changes in fluxes from dung-free pasture areas and dung beetle contribution had relatively little influence on the results (Fig. S2). Although changes in the effect of dung beetles had a proportionally large influence on the life cycle emissions from milk and beef production, the total effect of the dung beetles in the LCA remained small: changing the dung beetle effect on fluxes at the dung pat scale from a 14.5% reduction (estimate from this field study) to 43% reduction only resulted in 0.21% and 0.36% reduction in the LCA emissions of milk and beef, respectively.

The annual N_2O fluxes at the pasture scale were the most sensitive to variation in the flux from the dung-free pasture area, compared to which all other sources of variation were minor (Fig. S.3). This is due to the magnitude of the N_2O flux from dung pats and from dung-free areas. As the dung-free areas are much larger (96% vs. 4%), they will dominate the overall fluxes. For CH_4, the pattern is the opposite, as dung pats are hotspots of CH_4 emission. Thus, emissions from pats play a larger role in the pasture CH_4 budget compared with the N_2O budget. Similar to the life cycle of CH_4 emissions, the N_2O emission also showed a large proportional response to variation in the effect of dung beetles. Nonetheless, the total effect remained small, as a reduction of 6% (three times the observed value of 2%) only resulted in reductions of 0.34% and 0.62% in LCA emissions from milk and beef, respectively.

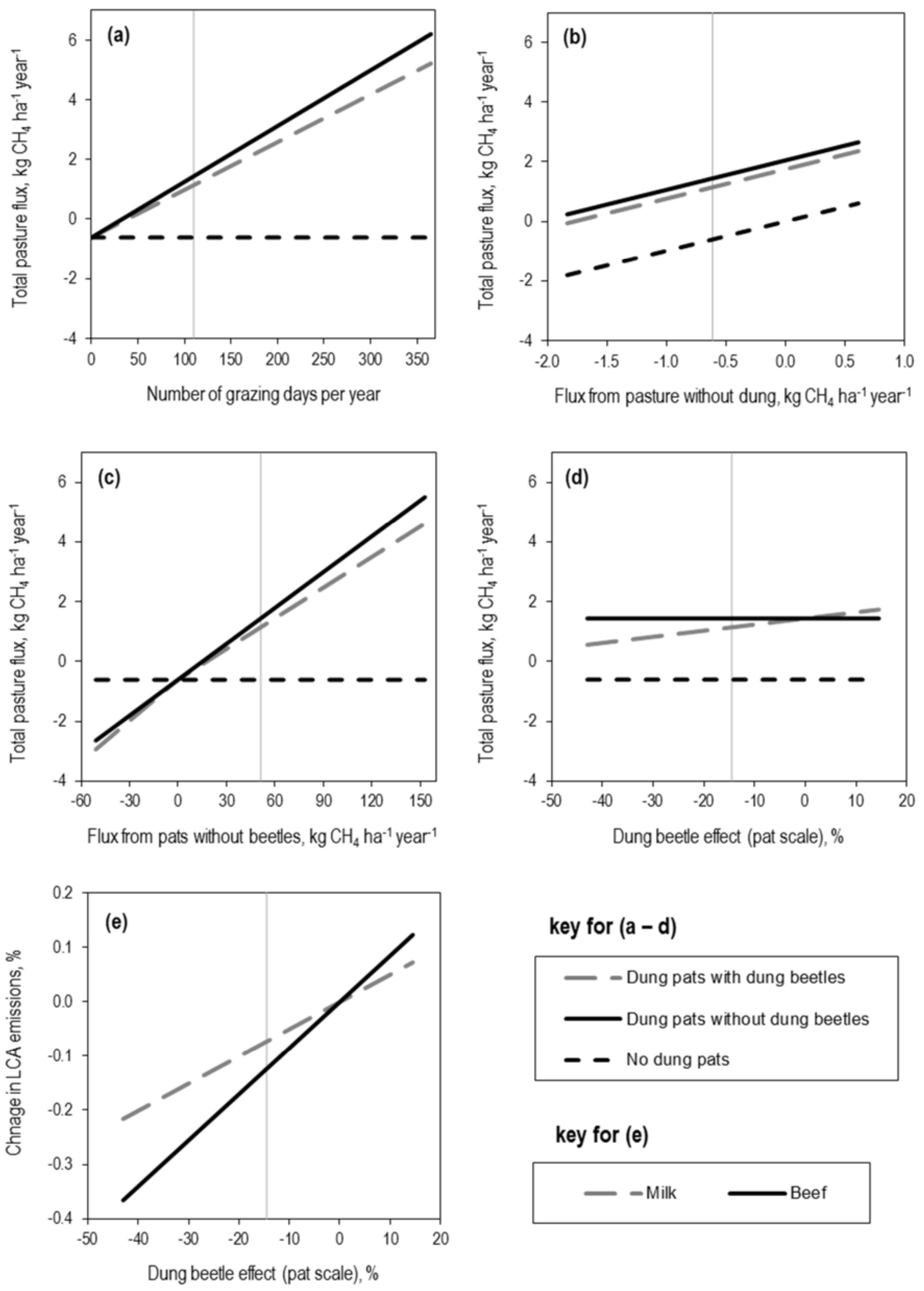
(a)
Total pasture flux, kg CH_4 ha^{-1} $year^{-1}$
Number of grazing days per year
(b)
Flux from pasture without dung, kg CH_4 ha^{-1} $year^{-1}$
(c)
Flux from pats without beetles, kg CH_4 ha^{-1} $year^{-1}$
(d)
Dung beetle effect (pat scale), %
(e)
Chnage in LCA emissions, %
key for (a – d)
Dung pats with dung beetles
Dung pats without dung beetles
No dung pats
key for (e)
Milk
Beef

Figure S.2. Sensitivity analysis of CH_4 flux (a-d) from the pasture ecosystem consisting of dung pats with dung beetles, dung pats without dung beetles, and pasture with no dung, and (e) during the life cycle of milk and beef production. Sensitivity of the pasture ecosystem flux estimates to (a) grazing season length, (b) changes in CH_4 flux from pasture areas without dung, (c) changes in CH_4 flux from dung, assuming a constant beetle effect at the pat scale, and (d) changing dung beetle effect at the pat scale. (e) Sensitivity of the life cycle CH_4 emissions (expressed as percent change of total emissions from milk and beef production) to changing dung beetle effect at the pat scale. The vertical grey lines denote the actual values in this study.

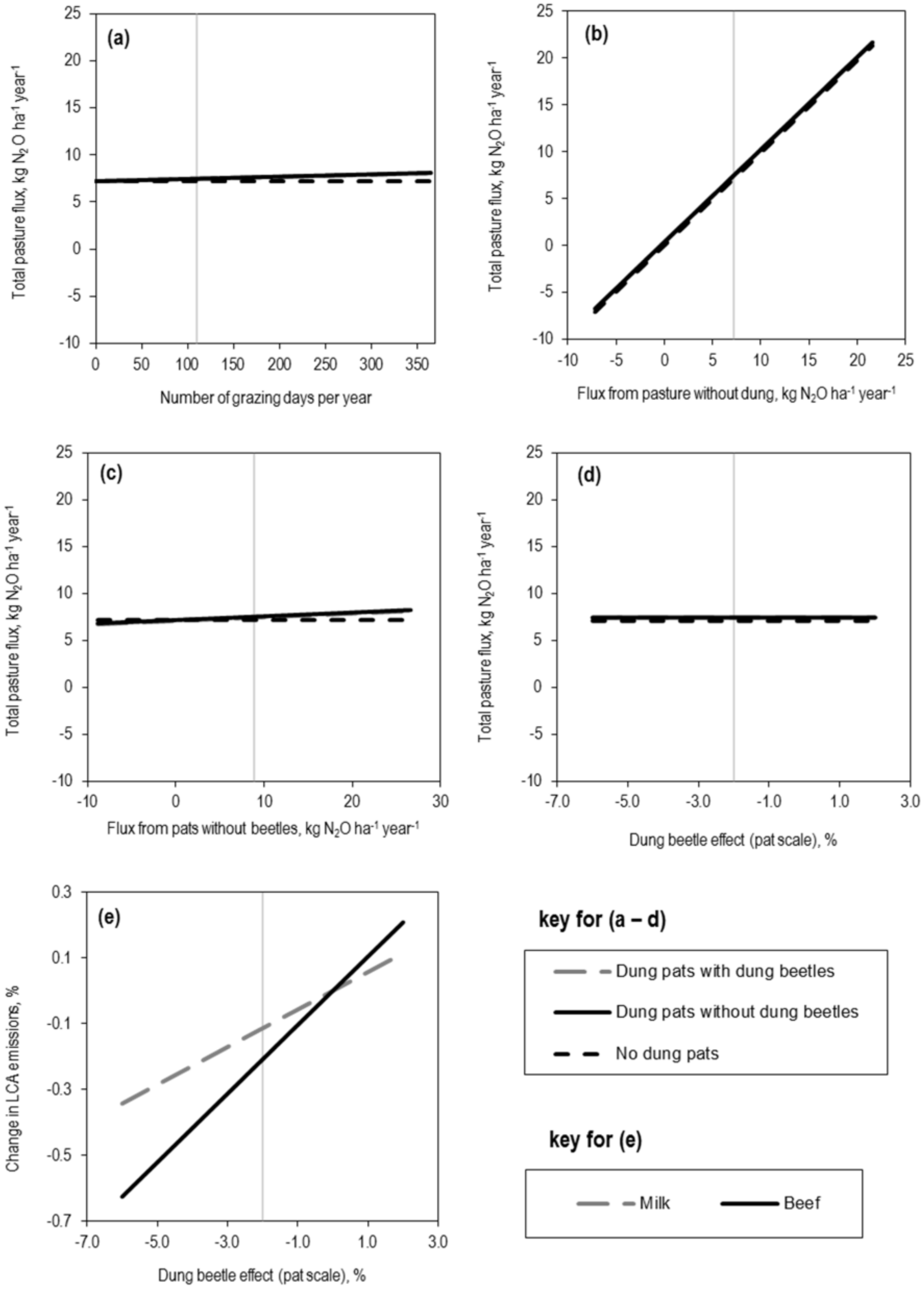
(a)
Total pasture flux, kg N_2O ha^{-1} year^{-1}
Number of grazing days per year
(b)
Flux from pasture without dung, kg N_2O ha^{-1} year^{-1}
(c)
Flux from pats without beetles, kg N_2O ha^{-1} year^{-1}
(d)
Dung beetle effect (pat scale), %
(e)
Change in LCA emissions, %
key for (a – d)
Dung pats with dung beetles
Dung pats without dung beetles
No dung pats
key for (e)
Milk
Beef

Figure S.3. Sensitivity analysis of N_2O flux (a-d) from the pasture ecosystem consisting of dung pats with dung beetles, dung pats without dung beetles, and pasture with no dung, and (e) during the life cycle of milk and beef production. Sensitivity of the pasture ecosystem flux estimates to (a) grazing season length, (b) changes in CH_4 flux from pasture areas without dung, (c) changes in N_2O flux from dung, assuming a constant beetle effect at the pat scale, and (d) changing dung beetle effect at the pat scale. (e) Sensitivity of life cycle N_2O emissions (expressed as percent change of total emissions from milk and beef production) to changing dung beetle effect at the pat scale. The vertical grey lines denote the actual values in this study.

References

1 Leip, A. *et al. Evaluation of the livestock sector's contribution to the EU greenhouse gas emissions (GGELS) - final report*. (European Commission, Joint Research Centre, 2010).
